# Tris(4,4′-bi-1,3-thia­zole-κ^2^ *N*,*N*′)iron(II) tetra­bromidoferrate(III) bromide

**DOI:** 10.1107/S1600536811004181

**Published:** 2011-02-09

**Authors:** Anita Abedi, Vahid Amani, Nasser Safari

**Affiliations:** a Department of Chemistry, Islamic Azad University, North Tehran Branch, Tehran, Iran; b Department of Chemistry, Shahid Beheshti University, G. C., Evin, Tehran 1983963113, Iran

## Abstract

In the [Fe(4,4′-bit)_3_]^2+^ (4,4′-bit is 4,4′-bi-1,3-thia­zole) cation of the title compound, [Fe(C_6_H_4_N_2_S_2_)_3_][FeBr_4_]Br, the Fe^II^ atom (3 symmetry) is six-coordinated in a distorted octa­hedral geometry by six N atoms from three 4,4′-bit ligands. In the [FeBr_4_]^−^ anion, the Fe^III^ atom (3 symmetry) is four-coordinated in a distorted tetra­hedral geometry. In the crystal, inter­molecular C—H⋯Br hydrogen bonds and Br⋯π inter­actions [Br⋯centroid distances = 3.562 (3) and 3.765 (2) Å] link the cations and anions, stabilizing the structure.

## Related literature

For general background to metal complexes with 4,4′-bi-1,3-thia­zole ligands, see: Baker & Goodwin (1985 ▶); Mahjoub & Morsali (2001 ▶, 2002*a* ▶,*b* ▶). For related structures, see: Al-Hashemi *et al.* (2009 ▶); Ali & Al-Far (2007 ▶); Amani *et al.* (2007*a* ▶,*b* ▶, 2009 ▶); Craig *et al.* (1988 ▶); Figgis *et al.* (1983 ▶); Jia *et al.* (2006 ▶); Khavasi *et al.* (2008 ▶); Kulkarni *et al.* (1998 ▶); Notash *et al.* (2008 ▶, 2009 ▶); Rahimi *et al.* (2009 ▶); Safari *et al.* (2009 ▶). For the synthesis of the ligand, see: Erlenmeyer & Ueberwasser (1939 ▶).
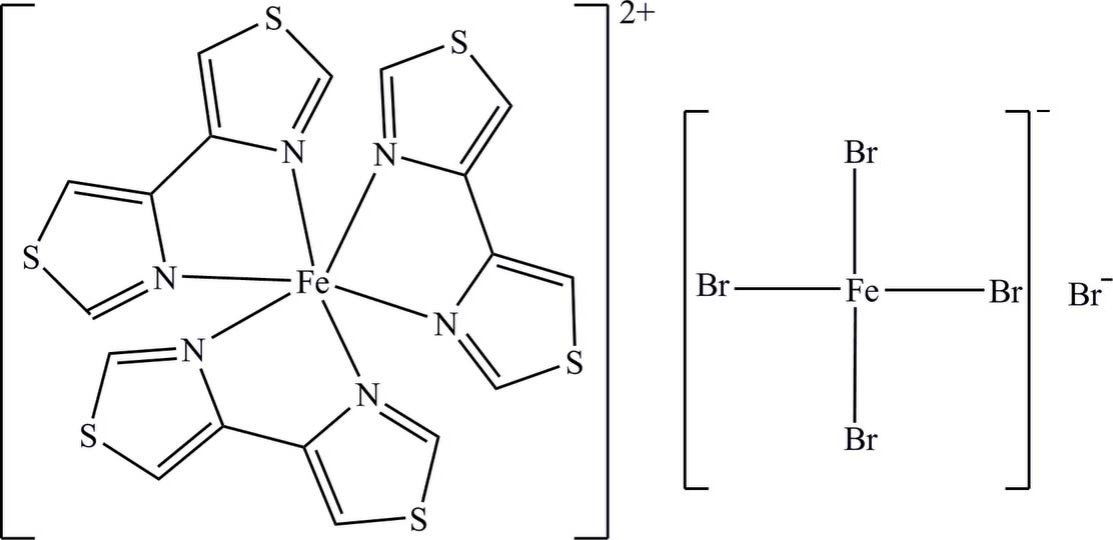


## Experimental

### 

#### Crystal data

[Fe(C_6_H_4_N_2_S_2_)_3_][FeBr_4_]Br
*M* *_r_* = 1015.95
Trigonal, 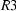

*a* = 12.0638 (7) Å
*c* = 17.6907 (13) Å
*V* = 2229.7 (2) Å^3^
*Z* = 3
Mo *K*α radiation
μ = 8.14 mm^−1^
*T* = 100 K
0.45 × 0.35 × 0.30 mm

#### Data collection

Bruker APEXII CCD diffractometer
Absorption correction: multi-scan (*SADABS*; Bruker, 2001 ▶) *T*_min_ = 0.031, *T*_max_ = 0.086
8430 measured reflections
2508 independent reflections
2427 reflections with *I* > 2σ(*I*)
*R*_int_ = 0.099

#### Refinement

*R*[*F* ^2^ > 2σ(*F* ^2^)] = 0.036
*wR*(*F* ^2^) = 0.085
*S* = 1.01
2508 reflections
112 parameters
1 restraint
H-atom parameters constrained
Δρ_max_ = 0.93 e Å^−3^
Δρ_min_ = −0.78 e Å^−3^
Absolute structure: Flack (1983 ▶), 1195 Friedel pairs
Flack parameter: 0.021 (9)

### 

Data collection: *APEX2* (Bruker, 2007 ▶); cell refinement: *SAINT* (Bruker, 2007 ▶); data reduction: *SAINT*; program(s) used to solve structure: *SHELXS97* (Sheldrick, 2008 ▶); program(s) used to refine structure: *SHELXL97* (Sheldrick, 2008 ▶); molecular graphics: *SHELXTL* (Sheldrick, 2008 ▶) and *Mercury* (Macrae *et al.*, 2006 ▶); software used to prepare material for publication: *SHELXTL*.

## Supplementary Material

Crystal structure: contains datablocks I, global. DOI: 10.1107/S1600536811004181/hy2404sup1.cif

Structure factors: contains datablocks I. DOI: 10.1107/S1600536811004181/hy2404Isup2.hkl

Additional supplementary materials:  crystallographic information; 3D view; checkCIF report

## Figures and Tables

**Table 1:** Selected bond lengths (Å)

Fe1—N1	1.962 (3)
Fe1—N2	1.974 (3)
Fe2—Br1	2.3348 (5)
Fe2—Br2	2.3370 (12)

**Table 2:** Hydrogen-bond geometry (Å, °)

*D*—H⋯*A*	*D*—H	H⋯*A*	*D*⋯*A*	*D*—H⋯*A*
C2—H2*A*⋯Br3	0.93	2.81	3.665 (5)	153
C5—H5*A*⋯Br3	0.93	2.97	3.798 (5)	149

**Fig. 1.**
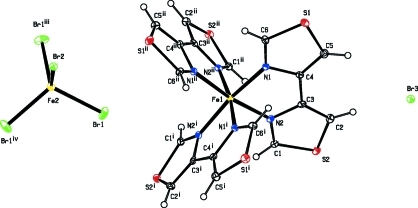
The molecular structure of the title compound. Displacement ellipsoids are drawn at the 30% probability level. [Symmetry codes: (i) 1-x+y, 2-x, z; (ii) 2-y, 1+x-y, z; (iii) 1-y, 1+x-y, z; (iv) -x+y, 1-x, z.]

**Fig. 2.**
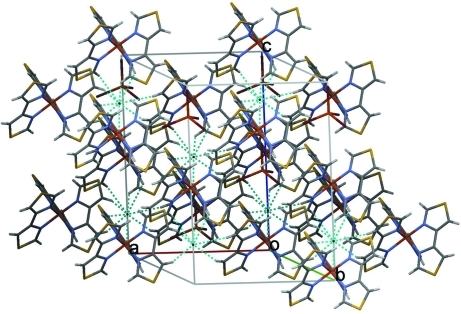
Crystal packing diagram for the title compound. Hydrogen bonds are shown as dashed lines.

**Fig. 3.**
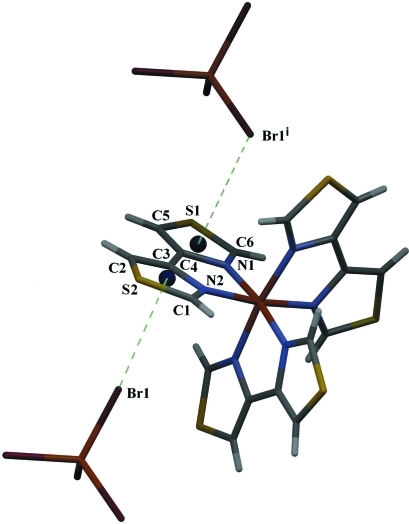
Intermolecular Br···π interactions (dashed lines) in the title compound. [Symmetry code: (i) 1-x+y, 2-x, z.]

## supplementary crystallographic information

### Comment

Erlenmeyer & Ueberwasser (1939) first reported the synthesis of 4,4'-bi-1,3-thiazole (4,4'-bit) and Craig *et al.* (1988) determined the structure of this compound. Although 4,4'-bit is a good bidentate ligand, a few of its metal complexes have been prepared, such as those of nickel and iron (Baker & Goodwin, 1985), lead (Mahjoub & Morsali, 2001, 2002*a*) and bismuth (Mahjoub & Morsali, 2002*b*). We recently introduced the coordination chemistry of 2,2'-dimethyl-4,4'-bi-1,3-thiazole with copper (Al-Hashemi *et al.*, 2009), zinc and mercury (Khavasi *et al.*, 2008; Safari *et al.*, 2009), cadmium (Notash *et al.*, 2009) and thallium (Notash *et al.*, 2008). We report here the synthesis and crystal structure of the title compound.

The asymmetric unit of the title compound (Fig. 1), contains one third of an [Fe(4,4'-bit)_3_]^2+^ cation, one third of an [FeBr_4_]^-^ anion and one third of a Br^-^ anion. In the [Fe(4,4'-bit)_3_]^2+^ cation, the Fe^II^ atom (3 symmetry) is six-coordinated in a distorted octahedral geometry by six N atoms from three 4,4'-bit ligands. The Fe—N bond lengths are 1.962 (3) and 1.974 (3) Å (Table 1). The average Fe—N bond distances in high-spin iron(II) and (III) complexes with phenanthroline and bipyridine are around 2.2 Å. However, for low-spin iron(II) and (III) complexes, the Fe—N distances less than 2.0 Å have been reported (Amani *et al.*, 2007*a*,b, 2009; Figgis *et al.*, 1983; Kulkarni *et al.*, 1998; Rahimi *et al.*, 2009). Therefore, in the [Fe(4,4'-bit)_3_]^2+^ cation, the Fe—N bond distances are unambiguous in accord with low-spin iron(II). The N—Fe—N bond angles are in the range of 82.00 (14) to 171.87 (14)°. The bond angles and distances are in good agreement to those of [Fe(4,4'-bit)_3_]^2+^ cations, which have been found in other structures (Baker & Goodwin, 1985). In the [FeBr_4_]^-^ anion, the Fe^III^ atom (3 symmetry) is four-coordinated in a distorted tetrahedral geometry by four Br atoms. The Fe—Br bond lengths are 2.3348 (5) and 2.3370 (12) Å. The Br—Fe—Br angles, in turn, span the ranges of 108.64 (3) to 110.29 (3)°, and the bond angles and distances are in good agreement to those of [FeBr_4_]^-^ anions, which have been found in other structures (Ali & Al-Far 2007; Jia *et al.*, 2006).

Fig. 2 shows significant intermolecular C—H···Br hydrogen bonds in the title compound (Table 2). The hydrogen bonds cause the formation of a supramolecular architecture, best described as built up by Br(thiazol)_9_ supramolecular synthons (Fig. 2) assembled *via* C—H···Br hydrogen bonds, where nine thiazole groups surround one (central) uncoordinated bromide ion. These synthons are further connected into an adamantoid-like network that extends into a three-dimensional structure. The discrete [FeBr_4_]^-^ anions occupy the cavities that result from the three-dimensional assembly of the Br(thiazol)_9_ entities. There also exist intermolecular Br···π interactions between the [FeBr_4_]^-^ anions and thiazole rings in the crystal structure (Fig. 3), with Br1···*Cg*1 = 3.562 (3) and Br1^i^···*Cg*2 = 3.765 (2) Å [*Cg*1 and *Cg*2 are the centroids of C1, C2, C3, N2, S2 ring and C4, C5, C6, N1, S1 ring. Symmetry code: (i) 1-x+y, 2-x, z]. The hydrogen bonds and Br···π interactions link the cations and anions, which may be effective in the stabilization of the structure.

### Experimental

4,4'-bi-1,3-thiazole (0.11 g, 0.63 mmol) in CH_3_OH (20 ml) was added to a solution of FeBr_3_ (0.06 g, 0.21 mmol) in CH_3_OH (10 ml) and the resulting red solution was stirred at 313 K for 1 h. The red colored precipitated product was recrystallized from CH_3_CN/CH_3_OH (v/v 2:1). After two weeks, dark-red prismatic crystals of the title compound were isolated (yield: 0.08 g, 75.0%; m.p. 464 K).

### Refinement

All H atoms were positioned geometrically and refined as riding atoms, with C—H = 0.93 Å and with *U*_iso_(H) = 1.2*U*_eq_(C).

### Crystal data

**Table:**

[Fe(C_6_H_4_N_2_S_2_)_3_][FeBr_4_]Br	*D*_x_ = 2.270 Mg m^−^^3^
*M**_r_* = 1015.95	Mo *K*α radiation, λ = 0.71073 Å
Trigonal, *R*3	Cell parameters from 1635 reflections
Hall symbol: R 3	θ = 3.0–28.0°
*a* = 12.0638 (7) Å	µ = 8.14 mm^−^^1^
*c* = 17.6907 (13) Å	*T* = 100 K
*V* = 2229.7 (2) Å^3^	Prism, dark-red
*Z* = 3	0.45 × 0.35 × 0.30 mm
*F*(000) = 1455	

### Data collection

**Table:**

Bruker APEXII CCD diffractometer	2508 independent reflections
Radiation source: fine-focus sealed tube	2427 reflections with *I* > 2σ(*I*)
graphite	*R*_int_ = 0.099
φ and ω scans	θ_max_ = 28.9°, θ_min_ = 2.3°
Absorption correction: multi-scan (*SADABS*; Bruker, 2001)	*h* = −16→16
*T*_min_ = 0.031, *T*_max_ = 0.086	*k* = −16→16
8430 measured reflections	*l* = −24→23

### Refinement

**Table:**

Refinement on *F*^2^	Secondary atom site location: difference Fourier map
Least-squares matrix: full	Hydrogen site location: inferred from neighbouring sites
*R*[*F*^2^ > 2σ(*F*^2^)] = 0.036	H-atom parameters constrained
*wR*(*F*^2^) = 0.085	*w* = 1/[σ^2^(*F*_o_^2^) + (0.0485*P*)^2^] where *P* = (*F*_o_^2^ + 2*F*_c_^2^)/3
*S* = 1.01	(Δ/σ)_max_ < 0.001
2508 reflections	Δρ_max_ = 0.93 e Å^−^^3^
112 parameters	Δρ_min_ = −0.78 e Å^−^^3^
1 restraint	Absolute structure: Flack (1983), 1195 Friedel pairs
Primary atom site location: structure-invariant direct methods	Flack parameter: 0.021 (9)

### Fractional atomic coordinates and isotropic or equivalent isotropic displacement parameters (Å^2^)

**Table:**

	*x*	*y*	*z*	*U*_iso_*/*U*_eq_	
Br1	0.53314 (4)	0.70585 (5)	0.46767 (3)	0.02946 (13)	
Br2	0.3333	0.6667	0.64196 (3)	0.01401 (14)	
Br3	1.6667	1.3333	0.53208 (4)	0.01483 (14)	
Fe1	1.0000	1.0000	0.48640 (5)	0.00941 (17)	
Fe2	0.3333	0.6667	0.50986 (6)	0.01351 (18)	
S1	1.25908 (10)	1.31480 (10)	0.64504 (6)	0.0182 (2)	
S2	1.33303 (9)	1.07879 (10)	0.33584 (6)	0.01552 (19)	
N1	1.1247 (3)	1.1426 (3)	0.54875 (19)	0.0120 (6)	
N2	1.1543 (3)	1.0443 (3)	0.42603 (19)	0.0121 (6)	
C1	1.1756 (4)	1.0038 (4)	0.3613 (2)	0.0141 (7)	
H1A	1.1107	0.9401	0.3323	0.017*	
C2	1.3758 (4)	1.1697 (4)	0.4169 (2)	0.0161 (7)	
H2A	1.4589	1.2296	0.4310	0.019*	
C3	1.2677 (4)	1.1394 (4)	0.4572 (2)	0.0132 (7)	
C4	1.2524 (4)	1.1950 (4)	0.5268 (2)	0.0127 (7)	
C5	1.3381 (4)	1.2906 (4)	0.5716 (3)	0.0185 (8)	
H5A	1.4262	1.3361	0.5639	0.022*	
C6	1.1159 (4)	1.1969 (4)	0.6109 (2)	0.0154 (7)	
H6A	1.0383	1.1732	0.6345	0.019*	

### Atomic displacement parameters (Å^2^)

**Table:**

	*U*^11^	*U*^22^	*U*^33^	*U*^12^	*U*^13^	*U*^23^
Br1	0.0213 (2)	0.0518 (3)	0.0176 (2)	0.0201 (2)	0.00229 (16)	−0.0064 (2)
Br2	0.01593 (18)	0.01593 (18)	0.0102 (3)	0.00796 (9)	0.000	0.000
Br3	0.01604 (19)	0.01604 (19)	0.0124 (3)	0.00802 (9)	0.000	0.000
Fe1	0.0096 (2)	0.0096 (2)	0.0090 (4)	0.00479 (11)	0.000	0.000
Fe2	0.0153 (3)	0.0153 (3)	0.0099 (4)	0.00764 (13)	0.000	0.000
S1	0.0184 (4)	0.0170 (4)	0.0170 (5)	0.0072 (4)	−0.0042 (4)	−0.0073 (4)
S2	0.0162 (4)	0.0198 (4)	0.0128 (4)	0.0107 (3)	0.0037 (3)	0.0013 (3)
N1	0.0113 (13)	0.0118 (14)	0.0122 (14)	0.0052 (12)	0.0016 (11)	0.0003 (11)
N2	0.0143 (14)	0.0143 (14)	0.0102 (13)	0.0090 (12)	0.0021 (12)	0.0016 (12)
C1	0.0135 (16)	0.0138 (16)	0.0157 (17)	0.0074 (14)	0.0000 (13)	−0.0008 (13)
C2	0.0161 (16)	0.0175 (17)	0.0139 (18)	0.0078 (14)	0.0027 (14)	0.0035 (14)
C3	0.0173 (17)	0.0131 (15)	0.0134 (16)	0.0106 (14)	0.0009 (13)	0.0036 (13)
C4	0.0156 (16)	0.0101 (15)	0.0126 (16)	0.0065 (13)	0.0010 (13)	0.0001 (12)
C5	0.0168 (17)	0.0198 (19)	0.0192 (19)	0.0092 (15)	−0.0020 (14)	−0.0002 (15)
C6	0.0135 (16)	0.0148 (17)	0.0151 (17)	0.0048 (13)	−0.0024 (14)	−0.0020 (13)

### Geometric parameters (Å, °)

**Table:**

Fe1—N1	1.962 (3)	N2—C1	1.320 (5)
Fe1—N2	1.974 (3)	N2—C3	1.385 (5)
Fe2—Br1	2.3348 (5)	C1—H1A	0.9300
Fe2—Br2	2.3370 (12)	C2—C3	1.366 (6)
S1—C6	1.706 (4)	C2—H2A	0.9300
S1—C5	1.721 (4)	C3—C4	1.458 (5)
S2—C1	1.706 (4)	C4—C5	1.355 (6)
S2—C2	1.720 (4)	C5—H5A	0.9300
N1—C6	1.312 (5)	C6—H6A	0.9300
N1—C4	1.396 (5)		
			
N1—Fe1—N1^i^	91.50 (14)	C6—N1—Fe1	133.8 (3)
N1—Fe1—N1^ii^	91.50 (14)	C4—N1—Fe1	115.4 (3)
N1^i^—Fe1—N1^ii^	91.50 (14)	C1—N2—C3	111.0 (3)
N1—Fe1—N2^i^	171.87 (13)	C1—N2—Fe1	134.5 (3)
N1^i^—Fe1—N2^i^	82.00 (14)	C3—N2—Fe1	114.6 (3)
N1^ii^—Fe1—N2^i^	93.53 (13)	N2—C1—S2	113.8 (3)
N1—Fe1—N2	82.00 (14)	N2—C1—H1A	123.1
N1^i^—Fe1—N2	93.53 (13)	S2—C1—H1A	123.1
N1^ii^—Fe1—N2	171.87 (13)	C3—C2—S2	108.8 (3)
N2^i^—Fe1—N2	93.49 (14)	C3—C2—H2A	125.6
N1—Fe1—N2^ii^	93.53 (13)	S2—C2—H2A	125.6
N1^i^—Fe1—N2^ii^	171.87 (14)	C2—C3—N2	115.4 (3)
N1^ii^—Fe1—N2^ii^	82.00 (14)	C2—C3—C4	129.9 (4)
N2^i^—Fe1—N2^ii^	93.49 (14)	N2—C3—C4	114.7 (3)
N2—Fe1—N2^ii^	93.49 (14)	C5—C4—N1	115.0 (4)
Br1^iii^—Fe2—Br1^iv^	110.29 (3)	C5—C4—C3	131.9 (4)
Br1^iii^—Fe2—Br1	110.29 (3)	N1—C4—C3	113.0 (3)
Br1^iv^—Fe2—Br1	110.29 (3)	C4—C5—S1	109.5 (3)
Br1^iii^—Fe2—Br2	108.64 (3)	C4—C5—H5A	125.2
Br1^iv^—Fe2—Br2	108.64 (3)	S1—C5—H5A	125.2
Br1—Fe2—Br2	108.64 (3)	N1—C6—S1	114.3 (3)
C6—S1—C5	90.4 (2)	N1—C6—H6A	122.8
C1—S2—C2	91.01 (19)	S1—C6—H6A	122.8
C6—N1—C4	110.7 (3)		
			
N1^i^—Fe1—N1—C6	−86.9 (3)	S2—C2—C3—N2	1.7 (4)
N1^ii^—Fe1—N1—C6	4.6 (4)	S2—C2—C3—C4	−175.8 (3)
N2—Fe1—N1—C6	179.8 (4)	C1—N2—C3—C2	−1.2 (5)
N2^ii^—Fe1—N1—C6	86.7 (4)	Fe1—N2—C3—C2	178.8 (3)
N1^i^—Fe1—N1—C4	88.3 (3)	C1—N2—C3—C4	176.7 (3)
N1^ii^—Fe1—N1—C4	179.8 (3)	Fe1—N2—C3—C4	−3.3 (4)
N2—Fe1—N1—C4	−5.1 (3)	C6—N1—C4—C5	−1.7 (5)
N2^ii^—Fe1—N1—C4	−98.1 (3)	Fe1—N1—C4—C5	−178.0 (3)
N1—Fe1—N2—C1	−175.5 (4)	C6—N1—C4—C3	−179.1 (3)
N1^i^—Fe1—N2—C1	93.5 (4)	Fe1—N1—C4—C3	4.7 (4)
N2^i^—Fe1—N2—C1	11.3 (4)	C2—C3—C4—C5	−0.2 (7)
N2^ii^—Fe1—N2—C1	−82.4 (3)	N2—C3—C4—C5	−177.7 (4)
N1—Fe1—N2—C3	4.6 (3)	C2—C3—C4—N1	176.7 (4)
N1^i^—Fe1—N2—C3	−86.5 (3)	N2—C3—C4—N1	−0.8 (5)
N2^i^—Fe1—N2—C3	−168.6 (3)	N1—C4—C5—S1	1.4 (5)
N2^ii^—Fe1—N2—C3	97.6 (3)	C3—C4—C5—S1	178.2 (3)
C3—N2—C1—S2	0.0 (4)	C6—S1—C5—C4	−0.6 (3)
Fe1—N2—C1—S2	−180.0 (2)	C4—N1—C6—S1	1.2 (4)
C2—S2—C1—N2	0.8 (3)	Fe1—N1—C6—S1	176.5 (2)
C1—S2—C2—C3	−1.4 (3)	C5—S1—C6—N1	−0.3 (3)

Symmetry codes: (i) −*x*+*y*+1, −*x*+2, *z*; (ii) −*y*+2, *x*−*y*+1, *z*; (iii) −*y*+1, *x*−*y*+1, *z*; (iv) −*x*+*y*, −*x*+1, *z*.

### Hydrogen-bond geometry (Å, °)

**Table:**

*D*—H···*A*	*D*—H	H···*A*	*D*···*A*	*D*—H···*A*
C2—H2A···Br3	0.93	2.81	3.665 (5)	153
C5—H5A···Br3	0.93	2.97	3.798 (5)	149
